# A Ubiquitination-Related Gene Prognostic Signature and the Oncogenic Role of RNF149 in Nasopharyngeal Carcinoma: scRNA-seq-Based Bioinformatics and Experimental Validation

**DOI:** 10.2174/0109298673378818250610053439

**Published:** 2025-06-25

**Authors:** Haiyan Deng, Juan Zhang, Shuaijun Chen, Tingfeng Liang, Xueyong Hu, Jing Li, Yong He, Feng Yu, Chaosheng Yu

**Affiliations:** 1 Department of Otorhinolaryngology-Head and Neck Surgery, Guangzhou Red Cross Hospital of Jinan University, Guangzhou, Guangdong, China;; 2 Department of Otorhinolaryngology-Head and Neck Surgery, Zhujiang Hospital, Southern Medical University, Guangzhou, China

**Keywords:** Nasopharyngeal carcinoma, scRNA-seq, ubiquitination, RNF149, prognostic signature, tumor organoids

## Abstract

**Introduction:**

Nasopharyngeal carcinoma (NPC) is an aggressive malignancy with a poor prognosis. Ubiquitination is a complex post translational modification involved in cancer progression. However, ubiquitination related genes (URGs) in immunotherapy of NPC remains largely unexplored.

**Methods:**

Differentially expressed URGs were screened based on the single-cell RNA sequencing (scRNA-seq) dataset and a risk model of NPC was constructed and evaluated for prognostic significance. The oncogenic role of RNF149 in NPC was investigated through *in vitro* and *in vivo* experiments, including tumor cells, NPC-like organoids, and tumor-bearing mice.

**Results:**

scRNA-seq data showed that URGs scores were higher in cancer cells than in normal epithelial cells. We identified 216 differentially expressed URGs between cancer and normal epithelial cells, but only 33 differentially expressed URGs associated with prognosis. Based on 33 URGs, TCGA-HNSC samples were classified into two distinct subtypes with significant differences in the tumor immune microenvironment, immunotherapy effect, and survival-prognostic genes. Using LASSO algorithm, 13 URGs were selected to construct a risk model, which demonstrated high predictive performance. The expression profiles of these 13 URGs were analyzed in TCGA-HNSC tumor and adjacent non-cancerous samples, and six URGs (BSPRY, OTUB1, PJA1, RNF149, RNF181, USP10) exhibited consistent expression trends. Moreover, quantitative real- time PCR revealed that RNF149 was up-regulated expression in NPC cells compared to the NP69 cells. RNF149 knockdown significantly impeded the proliferative, migratory, and invasive capabilities and exaggerated apoptosis of NPC cells. RNF149 knockdown cells exhibited a reduced capacity to form NPC organoids in a 3D culture system. shRNA-RNF149 diminished subcutaneous tumorigenic capacity of HK-1 cells compared to the control group.

**Discussion:**

The URGs-based prognostic risk model offers a robust tool for predicting immunotherapy efficacy in NPC and RNF149 promotes NPC progression.

**Conclusion:**

A URGs-related prognostic risk model capable of predicting clinical outcomes in NPC patients and RNF149 promotes NPC progression. Our findings are expected to provide new strategies to improve outcomes for NPC patients.

## 1 INTRODUCTION

Nasopharyngeal carcinoma (NPC), a rare malignant neoplasm arising from the nasopharyngeal epithelium, exhibits a high incidence rate, accounting for 83.3% of global cases, particularly in Southern and Southeast China populations [1, 2]. The early symptoms of NPC are generally not obvious, but NPC has a strong ability to invasion and metastasis. Currently, the mechanisms involved in the development and prognostic of NPC are still unclear. Previous studies have reported that multiple factors such as genetics, diet, viral infection, and smoking are involved in the tumorigenesis of NPC [3]. Due to lifestyle changes and advancements in diagnostic and therapeutic strategies, disease control and overall survival (OS) of NPC patients have been significantly improved [4, 5]. Recently, immunotherapy has emerged as a promising treatment. More and more clinical studies have explored the safety and efficacy of immunotherapy for advanced NPC, but there are still many patients who do not benefit from immunotherapy [6, 7]. Thus, it is essential to develop effective biomarkers to predict the efficacy of immunotherapy of NPC.

Ubiquitination is an important post-translational modification that regulates various key cellular processes. Several studies have shown that intracellular ubiquitination systems may contribute to the carcinogenesis process through regulation of cell cycle regulation, metabolic pathway regulation and DNA damage response [8-10]. The E3 ubiquitin ligase is a key member of the ubiquitin proteasome system, which identifies various oncogenic and tumor-suppressor factors and determines their fates through ubiquitination. Several studies have shown that the E3 ubiquitin ligases are aberrantly expressed and mutants in NPC, and these alterations are closely related to the development of NPC [11, 12]. For instance, E3 ubiquitin ligase TRIM21 suppresses mitochondrial DNA release and blocks antitumour immunity in NPC [13]. Deubiquitinase USP7 promotes cisplatin resistance and NPC progression [14]. In addition, DNA hypermethylation is significantly associated with E3 ubiquitin ligases, leading to increased radioresistance and enhanced tumor metastasis in NPC [15, 16]. These studies underscore the critical role of ubiquitination in NPC. Moreover, ring finger protein 149 (RNF149), an E3 ubiquitin ligase, has been reported to promote cancer progression, such as hepatocellular carcinoma [17] and esophageal squamous cell carcinoma [18]; however, its role in NPC remains unreported. Although researchers have focused on the function of ubiquitin ligases in NPC, the key genes regulated by ubiquitin ligases in NPC are not fully understood and require further exploration.

Integrated single-cell RNA sequencing (scRNA-seq) and bulk RNA-seq have great value in identifying biomarkers of diseases [19]. Our research aimed to identify potential ubiquitination-related genes (URGs) as effective biomarkers for predicting immunotherapy response in NPC. Studying the prognostic and immunotherapy effects of NPC and excavating key genes may help to promote the identification of new therapeutic targets, which is essential for subsequent gene-targeted therapy and improving the prognosis of NPC patients.

## 2 MATERIALS AND METHODS

### 2.1 Data Sources

The gene sets related to ubiquitination from the Molecular Signatures Database (https://www.gsea-msigdb.org/gsea/msigdb) and 488 URGs were downloaded (REACTOME_PROTEIN_UBIQUITINATION, GOMF_UBIQUITIN_LIKE_MODIFIER_ACTIVATING_ENZYME_ ACTIVITY, GOCC_UBIQUITIN_CONJUGATING_ENZYME_COMPLEX, GOCC_UBIQUITIN_LIGASE_COMPLEX). The GSE150430 dataset was selected based on a systematic search of the Gene Expression Omnibus (GEO) (http://www. ncbi.nlm.nih.gov/geo/) database using the following criteria: (1) Organism: Homo sapiens, (2) Data type: Expression profiling by high-throughput sequencing, (3) Disease: Nasopharyngeal carcinoma (NPC), and (4) Sample type: Tissue. This search yielded 36 datasets, which were further screened to exclude datasets containing Epstein-Barr virus (EBV) infection, exosome-related studies, and overexpression models, as these factors may introduce confounding variables unrelated to our study objectives. After applying these exclusion criteria, GSE150430 was chosen as it includes single-cell RNA sequencing (scRNA-seq) data from 15 NPC patients and 1 comparable sample, making it a suitable dataset for our bioinformatics analysis. The Cancer Genome Atlas - head and neck squamous cell carcinoma (TCGA-HNSC) data (n=500) were downloaded from the Genomic Data Commons (https://portal.gdc.cancer.gov/).

To further validate the robustness and generalizability of our URG-based prognostic risk model, we utilized the independent GSE102349 dataset, which includes 113 NPC patients with transcriptomic data and progression-free survival (PFS) data for 88 patients. Transcripts per million expression data for 113 NPC patients were downloaded from the GEO database (GSE102349). To account for batch effects, we applied the Combat algorithm to normalize the expression profiles using TCGA-HNSC tumor samples as a reference.

### 2.2 Data Analysis of scRNA-seq

The scRNA-seq dataset GSE150430 was analyzed using the Seurat package (v4.3.0.1). After importing the gene count matrix into the Seurat package (v4.3.0.1), the mitochondrial gene expression intensity in each cell was first calculated. Cells with mitochondrial gene expression exceeding 4% were removed. The cell data were normalized using the NormalizeData function. Based on this, the samples were batch-corrected using Seruat software. Principal component analysis was performed using batter-corrected data. The sorted cells were then visualized using the UMAP method. The BlueprintEncodeData dataset in the SingleR package (v1.6.1) and celldex package (v1.2.0) were subsequently used for cell annotation. Known cell marker genes were used to validate and refine the cell annotation results. The FindAllMarkers function was used to identify differentially expressed genes (DEGs) in each type of cell and to select DEGs between groups. The InferCNV (copy number variation) package (v1.42.2) was used to calculate CNVs in individual cells. The data of CNVs were used to judge malignant and normal epithelial cells. The scores of KEGG pathways for each cell subtype were calculated using the Gene Set Variation Analysis (GSVA) package (v1.46.0) and the msigdbr package (v7.5.1), and heatmaps were drawn using pheatmap (v1.0.12). The scores of ubiquitin-related genes (URGs) and ubiquitinated subset genes (E1, E2, E3, and deubiquitinating enzymes (DUBs)) in individual cells were calculated using the AUCell package (v1.20.2). To determine the threshold for defining cells with active gene sets, we applied the AUCell function aucThresholds, which identifies an optimal cutoff based on the distribution of area under curve (AUC) scores. Specifically, the threshold was automatically defined as the inflection point of the AUC distribution curve where the first derivative reaches its maximum, which corresponds to the transition point between the bimodal distribution of 'active' and 'inactive' cell populations. Violin plots were drawn using the ggplot2 package (v3.4.2) to show the differences in scores across each cell subtype.

### 2.3 Kaplan-Meier Analysis

Utilizing the Kaplan-Meier Plotter (http://kmplot.com), an online resource, we executed a survival analysis to elucidate the prognostic disparities between distinct cluster subtypes. The survival curves were juxtaposed through the application of the Kaplan-Meier estimator. Subsequently, the log-rank test was employed to statistically evaluate the observed differences in survival outcomes.

### 2.4 Analysis of Gene Set Variation

Pathway analysis was performed primarily to assess the activation of marker and metabolic pathways. Pathway activity estimates were assigned to individual cells with the use of GSVA in the GSVA package (v1.26.0).

### 2.5 Immune Cell Infiltration Analysis

The CIBERSORT R package was used to analyze the immune infiltration of 22 kinds of immune cells in different types of samples. The Wilcoxon test was used to detect differences in immune cell abundance. Spearman analysis was utilized to explore the correlation between 22 immune cell types.

### 2.6 Cell Culture and Transfection

NPC/HK-1 (iCell Bioscience, iCell-h367) cells, NP69 cells (iCell Bioscience, iCell-h431), and NPC/C666-1 cells (iCell Bioscience, iCell-h378) were cultured at Roswell Park Memorial Institute 1640 medium (Corning, 10-040-CVRC) + 10% fetal bovine serum (10099-141, GIBCO) + 1%PS in 37°C, 5%CO_2_. RNF149-siRNA-1/2/3 (Generay, Shanghai) and si-NC were transfected into NPC/HK-1 cells using Lipofectamine™ 2000 (Invitrogen) transfection reagent. The siRNA sequences are listed in Supplementary Table **1**. To stably express RNF149 in NPC/HK-1 cells, stable lentiviral transfection of pSLenti-U6-shRNA (RNF149)-CMV-EGFP-F2A-Puro-WPRE for HK-1 cells was performed.

### 2.7 RNA Extraction and Quantitative Real-time PCR (qPCR)

Cellular RNA was extracted with TRIzol reagent (Invitrogen, America), and RNA was used to reverse transcribe first-strand cDNA and then synthesize double-strand cDNA using a cDNA synthesis kit (#K1622, Thermo, China). qPCR analysis was performed according to the instructions for FastStart Universal SYBR Green Master Mix. All primer sequences are listed in Supplementary Table **1**.

### 2.8 Gene Set Enrichment Analysis (GSEA)

Gene set enrichment analysis (GSEA) was performed among the NPC samples of GSE150430 datasets. The normalized expression data were uploaded into GSEA 4.3.2 software for analysis. A normalized enrichment score (NES) greater than 1 and a false discovery rate of less than 0.25 were used to assess the magnitude of enrichment and statistical significance [20].

### 2.9 Cell Count Kit (CCK)-8 Experiment

Cultured cells were digested using Trypsin (Trypsin-EDTA) (11668-500, Invitrogen) to make single-cell suspensions. After counting, each group of cells was paired to 1 × 10^4^ cells / mL. Each well was spread with 100 μl cells (1000 cells / well). The well plate was incubated at 37°C and 5% CO_2_. Before detection, 10 μL CCK-8 (Beyotime, Shanghai) was added. After 1 h, the optical density value was measured at 450 nm on a microplate reader (Infinite M1000, TECAN).

### 2.10 Transwell Assays

NPC cell lines were seeded in Transwell chambers containing 200 μl serum-free medium. The invasion ability of the cells was tested by coating the surface of the upper chamber of the transwell chamber with a matrigel mixture. It is not necessary to coat the bottom of the chamber with Matrigel when examining the migration ability of the cells. The medium containing 10% fetal bovine serum was added and incubated overnight. Then the chamber was stained with crystal violet, 5 randomly selected fields were photographed, and cells were counted under a microscope.

### 2.11 Flow Cytometry

After transfecting with siNC or siRNF149 for 6 hours, the medium was replaced with fresh culture medium, and then HK-1 cells were collected after 48 hours for flow cytometry to assess apoptosis using Annexin V-FITC Cell Apoptosis Detection Kit (E606336-0100, Sangon, Shanghai, China). The cells were resuspended in 195 μL of 1X Binding Buffer to achieve a cell density of 2-5 × 10^5^ cells/mL. Subsequently, 5 μL of Annexin V-FITC was added, and the mixture was incubated at room temperature for 15 minutes. After washing, the cells were centrifuged at 1000 rpm for 5 minutes and then resuspended in 190 μL of 1X Binding Buffer, followed by adding 10 μL of Propidium Iodide. Flow cytometric analysis was completed within 4 hours on a flow cytometer (Beckman-Coulter).

### 2.12 NPC Organoids Induction

NPC tumor tissues were collected from the Guangzhou Red Cross Hospital of Jinan University and approved by the Ethics Committee of Zhujiang Hospital of Southern Medical University (2025-KY-031-01). All patients signed the informed consent form. Clinical information for the study participants is given in Table **1**. NPC tumor tissues were placed in pre-cooled 1× phosphate buffer solution (PBS) buffer at 4°C in a 10 cm culture dish and cut into tissue fragments of 1-3 mm^3^ following washed with PBS three times. The fragments were then transferred to a dissociation solution (100 U/mL collagenase, 125 U/mL deoxyribonuclease, and 2 mM CaCl_2_ in RPMI-1640) for incubation with shaking (37°C, 5% CO_2_, 75 RPM, 25 min). 40 U of papain was added to the dissociation solution to achieve a final papain concentration of 10 U/mL and incubation was continued with agitation. The resulting suspension was sequentially filtered through 100 μm and 40 μm cell strainers, followed by centrifugation at 250 g for 3 minutes at 4°C to collect the cells. The cell suspension was mixed with Matrigel at a 1:1 ratio and embedded. A total of 30 μL per well was seeded at the center of the well plate and immediately transferred to a cell incubator for static incubation for 15 minutes to allow the Matrigel to solidify, after which 500 μL of complete culture medium was added. After 5-7 days of culture, the culture medium was aspirated using a pipette, and 1-2 mL of pre-cooled (4°C) PBS was added to each well and left for 2 minutes. The Matrigel was gently pipetted to facilitate dissociation and collected into a 15 mL centrifuge tube, followed by static incubation at 4°C for 10 minutes. The suspension was centrifuged at 300 g for 5 minutes, the supernatant was discarded, and an appropriate amount of organoid digestion solution was added for digestion for 2-3 minutes. The suspension was then centrifuged at 300 g for 5 minutes, the supernatant was discarded. Next, the filtration and embedding steps were repeated. According to the experimental groups, 10 μL of lentiviral (shNC or shRNF149) and polybrene at a final concentration of 5 μg/mL were added into cultures. The cultures were incubated at 37°C for 24 hours, followed by replacing the medium with fresh culture medium. The growth of organoids was observed under a microscope (OL YMPUS, CKX53).

### 2.13 Animal Study

The tumor-forming experiment was approved by Jinan University Laboratory Animal Ethics Committee (LAEC-2023-261). A total of 10 female BALB/C nude mice (4-6 weeks old, 16-18g, SPF grade) were purchased from Beijing SPF Biotechnology Co., Ltd. The mice were acclimated for 7 days under conditions of 24-26°C, 55-60% humidity, and a 12-hour light/dark cycle. Mice were provided ad libitum access to a water and standard diet, specifically the ^60Co-irradiated maintenance feed for rats and mice (Cat# SPF-F02-002) provided by Speifu (Beijing) Biotechnology Co., Ltd. This diet comprised corn, soybean meal, fish meal, calcium hydrogen phosphate, various vitamins, multiple trace elements, and amino acids. To establish a tumor-bearing mouse model, 50 μL of cell suspension was mixed with 50 μL of Matrigel (Corning, 356230). The mixture was injected subcutaneously into each mouse at a density of 5 × 10^6^ cells per mouse, with the axilla chosen as the injection site. Tumor size was measured every 3 days starting 7 days after cell inoculation. Tumor volume was calculated using the formula: V = 1/2 length × width × width. On day 13 post-inoculation, mice were euthanized *via* cervical dislocation, and the tumor tissues were excised.

### 2.14 Hematoxylin and Eosin (H&E) Staining

H&E staining was performed to assess the histopathological characteristics of tumor tissues. Briefly, tumor specimens were fixed in 10% neutral-buffered formalin, dehydrated through a graded ethanol series, embedded in paraffin, and sectioned at 4 μm thickness. The sections were then deparaffinized with xylene, rehydrated with descending ethanol concentrations, and stained with hematoxylin to visualize cell nuclei, followed by eosin counterstaining to highlight cytoplasmic and extracellular matrix components. After dehydration and clearing, the stained sections were mounted and examined under a light microscope for histopathological evaluation.

### 2.15 Statistical Analysis

All data are recorded as the mean ± standard error of mean. Statistical analysis was performed using GraphPad Prism (v8.0) (San Diego, USA). An unpaired two-tailed t test was used. The threshold for statistical significance was set at *p* < 0.05.

## 3 RESULTS

### 3.1 URGs Mainly Expressed in Malignant Epithelial Cells in scRNA-seq Data

**Fig. (2):**
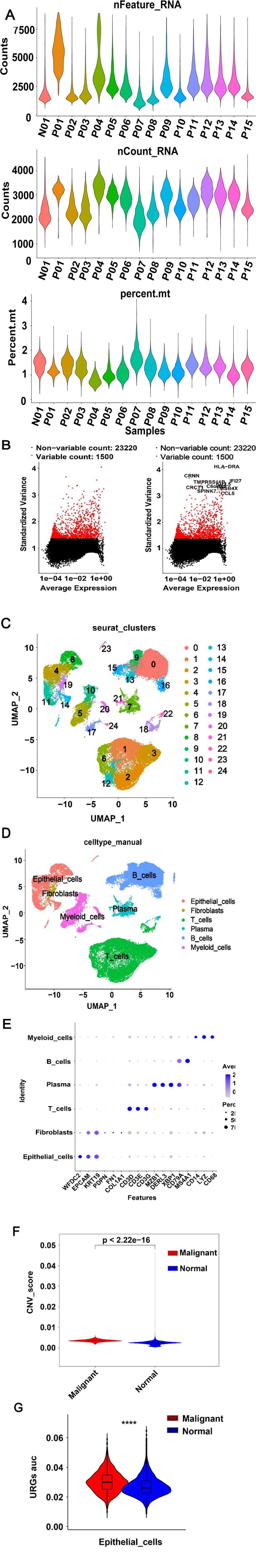
Identification of cell subtypes. (**A**) Quality control metrics of scRNA-seq data, including the number of detected features (nFeature), the number of unique molecular identifiers (nCount), and the percentage of mitochondrial gene expression (percent.mt) across individual cells. (**B**) Scatter plot illustrating the standard deviation of variable genes, which were selected for downstream analysis based on their variability across cells. (**C**) The t-SNE algorithm was applied to visualize the 25 cell clusters. (**D**) The UMAP algorithm identified the six cell types. (**E)** Bubble plots showing the expression levels of cell marker genes in six cell types. (**F**) Copy number variation (CNV) score divided epithelial cells into normal and tumor groups. (**G**) URGscore of tumor and normal epithelial cells. *** *p* < 0.001.

The workflow of this study is illustrated in Fig. (**1**). To investigate the predominant cell types expressing URGs, we characterized the expression profiles of 488 URGs using a scRNA-seq dataset GSE150430. Firstly, quality control was performed on the scRNA-seq data. After quality control, 48,584 cells were retained (Fig. **2A**). Utilizing the standardized data set, we have selected the 1500 genes exhibiting the most significant fluctuations (Fig. **2B**). Subsequently, the t-SNE algorithm was applied to identify cells into 25 distinct cell clusters (Fig. **2C**). Following this, an annotation process was conducted to determine the cell identity within each cluster, revealing the presence of six unique cell subtypes (Fibroblasts, Epithelial_cells, T_cells, Plasma, B_cells, Myeloid_cells) (Fig. **2D**). Besides, the expression of the marker gene of the cell subtype was displayed by bubble plots (Fig. **2E**). Among them, LYZ and CD68 were specific makers for Myeloid_cells, MS4A1 for B_cells, MZB1, DERL3 and XBP1 for Plasma, and CD3D, CD3E and CD3G for T_cells (Fig. **2E**).

Ubiquitination refers to the process of covalent binding of ubiquitin molecules to target proteins catalyzed by enzymes [21]. Ubiquitination modification plays a significant role in various biological processes [22, 23], but its role in NPC still needs further investigation. Next, to determine the distribution of ubiquitination levels in different cells, we calculated the scores of E1, E2, E3, and DUBs in different cells. We found that the E2 ubiquitin ligase score was highest in epithelial cells (Supplementary Fig. **1A**). Subsequently, inferCNV was used to distinguish tumor cells and normal epithelial cells in epithelial cells. CNV score analysis was performed on epithelial cells from all samples (Supplementary Fig. **1B**). Epithelial cells were successfully divided into normal and tumor groups (Fig. **2F**). As expected, we found that the URGscore of tumor cells was significantly higher than normal epithelial cells in the scRNA-seq dataset (Fig. **2G**). In summary, URGs are predominantly expressed in tumor cells.

### 3.2 Identification of URGs Related Subtypes and Characterization of Immune Characteristics

**Fig. (3):**
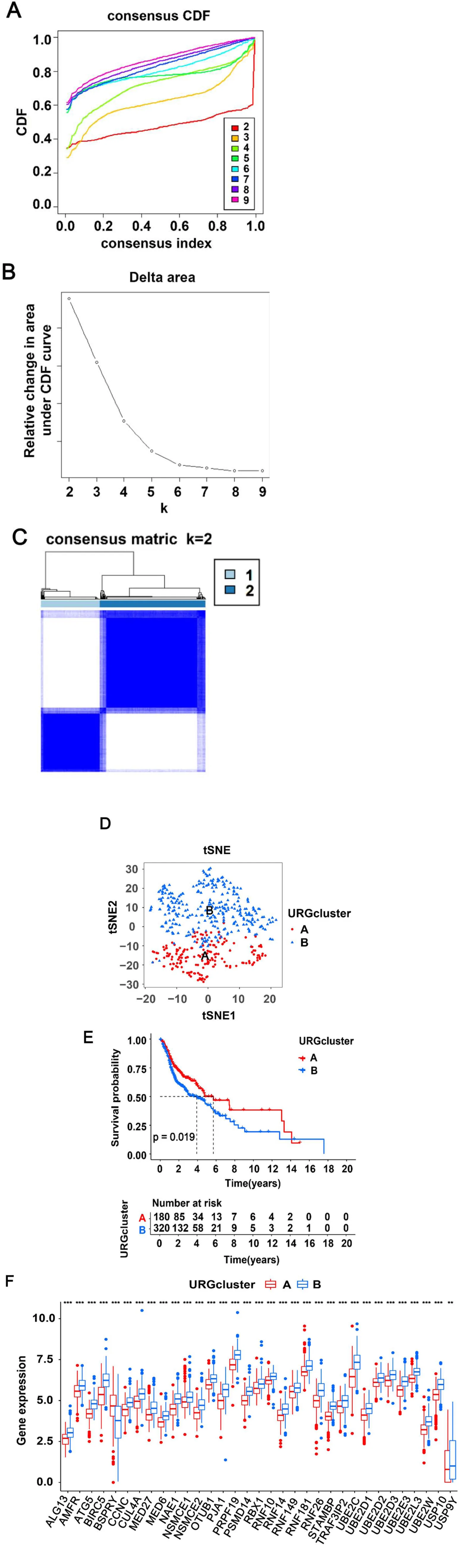
Identification of 33 differentially expressed URGs related cluster. (**A**) Consistency clustering cumulative distribution function (CDF) curves for K = 2 - 9, illustrating the stability of clustering results across different configurations. (**B**) Relative changes in the area under the CDF curve when K = 2 - 9. (**C**) Heatmap of clustering results for K=2, visualizing the similarity between data points within the clusters. (**D**) The tSNE plot showed that the two subtypes were clearly typed. (**E**) Kaplan-Meier survival curves for the URGcluster A and URGcluster B. (**F**) URGs expression in the URGcluster A and URGcluster B. *** *p* < 0.001, ** *p* < 0.01.

To identify NPC-related URGs, differentially expressed URGs between tumor and normal epithelial cells were identified from the scRNA-seq dataset, and a total of 216 differentially expressed URGs were obtained (false discovery rate < 0.05) (Supplementary Fig. **2A**), but only 33 prognostic-related genes passed unicox analysis with 95% confidence intervals (*p*<0.05, Supplementary Figs. **2B**-**C**). Next, consensus clustering analysis was performed based on these 33 differentially expressed URGs on TCGA-HNSC samples using the R package “ConsensusClusterPlus” (Figs. **3A**-**C**). Consensus matrix heatmap implicated that the optimal value was K = 2 (Fig. **3C**), and the tSNE plot demonstrated that the NPC patients were distinctly clustered into two subtypes (Fig. **3D**). Compared to the URGcluster B, patients in the URGcluster A cluster exhibited significantly longer survival time (*p*=0.019, Fig. **3E**). Furthermore, (Fig. **3F**) shows that 33 URGs were differentially expressed between URGcluster A and URGcluster B. Among them, compared to URGcluster A, 32 URGs were up-regulated in URGcluster B, and BSPRY was down-regulated in URGcluster B Fig. (**3F**). These results indicate that the 33 differentially expressed URGs are closely associated with NPC.

**Fig. (4):**
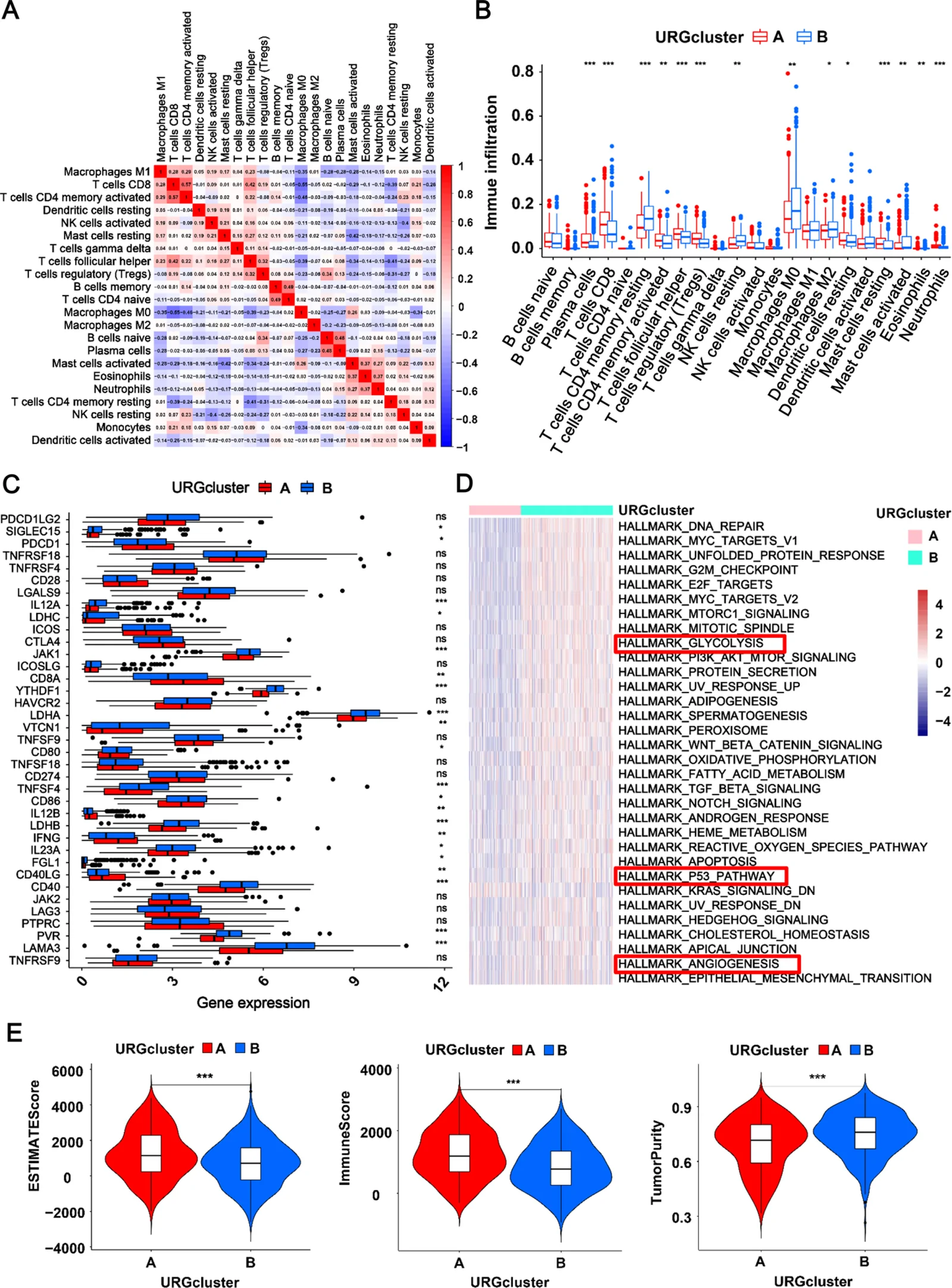
Characterization of immunologic characteristics between two subtypes. (**A**) Correlation of 22 immune cell types. The red and blue colors represent positive and negative correlations, respectively. (**B**) Comparison of immune cell abundance of NPC samples between URGcluster A and URGcluster B. (**C**) Expression of immune checkpoint-related genes in NPC samples between URGcluster A and URGcluster B samples. (**D**) Comparison of functions and pathways between URGcluster A and URGcluster B using GSVA. (**E**) ESTIMATE score, immunoscore and tumor purity between two subtypes. *** *p* < 0.001, ** *p* < 0.01, * *p* < 0.05.

To investigate whether these 33 differentially expressed URGs are associated with the tumor immune microenvironment of NPC, we performed an immune cell infiltration analysis. We found that, among the 22 immune cells, T cells CD8 was positively correlated with T cells CD4 memory activated (r = 0.57) and T cells follicular helper (r = 0.42) (Fig. **4A**). Meanwhile, T cells CD8 was negatively correlated with macrophages M0 (r = -0.55) and T cells CD4 memory resting (r = -0.39) (Fig. **4A**). Assessment of immune infiltration between URGcluster A and URGcluster B has uncovered substantial disparities in the presence of 14 distinct immune cell types between the two groups (Fig. **4B**). The abundance of tumor-killing CD8 T cells was increased in low-risk URGcluster A, and the abundance of immunosuppressive M2 macrophages was increased in high-risk URGcluster B (Fig. **4B**). The limma package showed that the expression of immune checkpoint-related genes was differentially expressed between URGcluster A and URGcluster B (Fig. **4C**). In contrast to URGcluster A, SIGLEC15, IL12A, LDHC, JAK1, TYHDF1, LDHA, VTCN1, CD80, TNFSF4, CD86, LDHB, IL23A, FGL1, CD40, PVR and LAMA3 were up-regulated in URGcluster B. PDCD1, CD8A, IL12B, IFNG, and CD40LG were downregulated in URGcluster B (Fig. **4C**). In addition, significant differences in pathway activation status were also observed between URGcluster A and URGcluster B (Fig. **4D**). We found that several pro-tumor-related pathways were activated in URGcluster B, such as p53, angiogenesis, and glycolysis (Fig. **4D**). In addition, the estimation of stromal and immune cells in malignant tumor tissues using expression data (ESTIMATE) score and immunity score in URGcluster B were significantly lower than URGcluster A, and the tumor purity in URGcluster B was significantly higher than URGcluster A (Fig. **4E**). In conclusion, subtypes based on the 33 differentially expressed URGs participate in different pathways and present different tumor immune microenvironment, which is of great significance for clinical practice and research in NPC.

### 3.3 Construction and Evaluation of URG-based Prognostic Risk Models

**Fig. (5):**
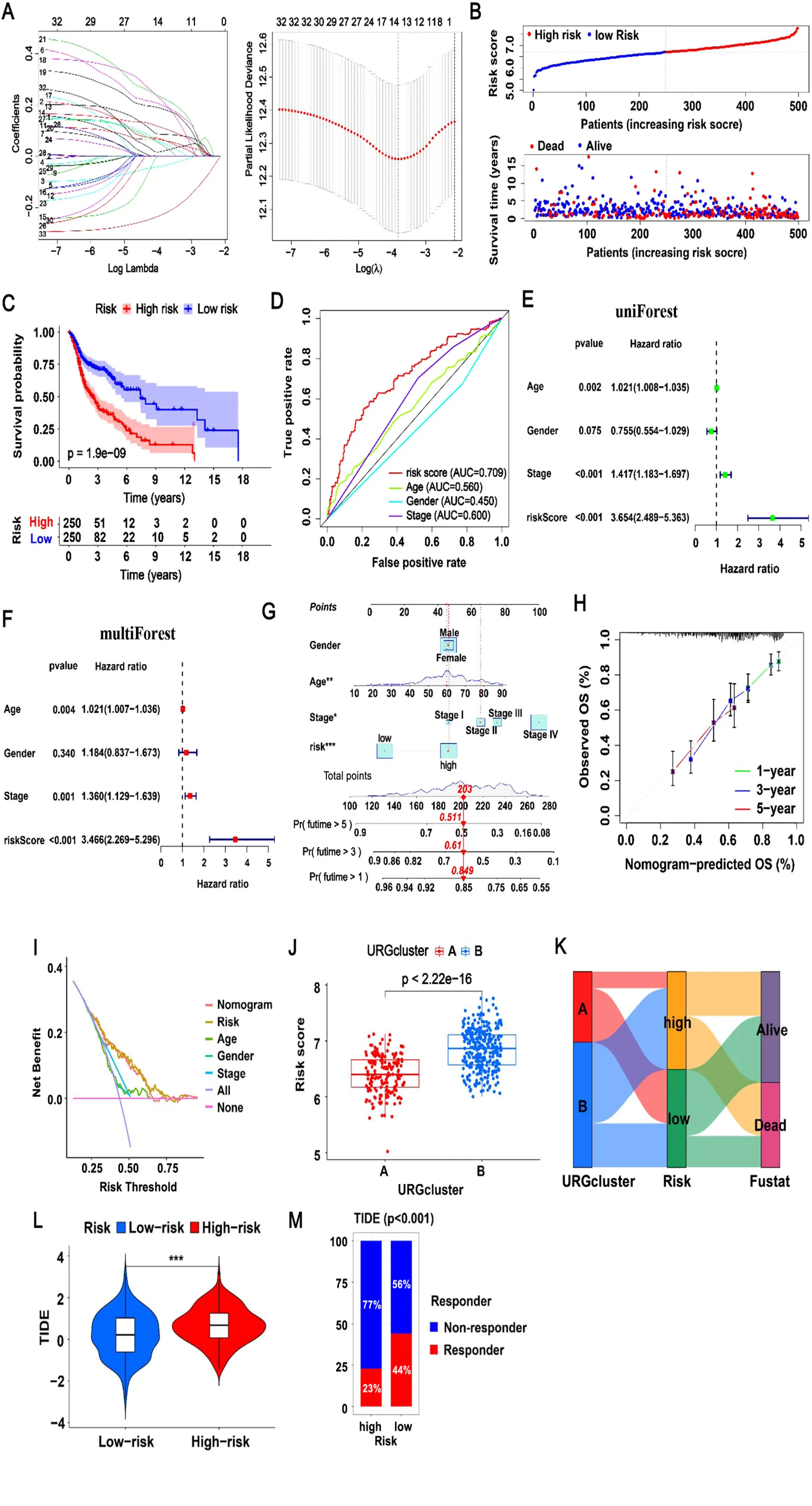
Construction and evaluation of prognostic risk models. (**A**) LASSO regression analysis further screened 13 URGs. Trajectories for each independent variable and confidence intervals under each lambda are shown. (**B**) Scatter plot illustrating the correlation between high- and low-risk samples and survival time. Red and blue dots represent deceased and surviving patients, respectively. (**C**) Kaplan-Meier analysis between different risk groups. (**D**) Receiver operating characteristic curve analysis comparing the predictive performance of the risk model with age, gender, and tumor stage. (**E**, **F**) Univariate and multivariate Cox analysis of risk score and clinical characteristics. Parentheses indicate the 95% confidence intervals for the hazard ratios. (**G**) Nomogram model based on URGs and clinical features for predicting 1-, 3-, and 5-year survival rates of NPC patients. (**H**) Calibration plots of concordance tests between 1-, 3-, and 5-year survival predictions and actual outcomes. (**I**) Decision curve analysis curves. Decision curve for 1-, 3-, and 5-year overall survival (OS) probability. (**J**) Boxplot shows the risk scores of the two cluster groups. (**K**) Sankey plot showing potential correlations between clustering, risk score, and survival status. **(L**) Tumor immune dysfunction and exclusion (TIDE) scores in the high-risk and low-risk groups. (**M**) Proportion of responses to immunotherapy in TIDE subtypes. *** *p* < 0.001.

The LASSO algorithm was used to construct the URG-based risk model. Fig. (**5A**) (left) presents the trajectory of coefficients for the candidate genes as the regularization parameter (log λ) varies. With increasing log λ, the coefficients of most genes gradually shrink toward zero, indicating the feature selection process. In Fig. (**5A**) (right), the partial likelihood deviance was plotted against log λ to determine the optimal tuning parameter *via* 10-fold cross-validation. The two vertical dashed lines represent the values of log λ corresponding to the minimum deviance and one standard error from the minimum. Specifically, the log λ values of -2 and -4 corresponded to 1 and 13 nonzero coefficients, respectively. To balance model complexity and predictive performance, we selected 13 genes at log λ = -4 for constructing the NPC prognostic risk model. Risk score = (0.06338707 × AMFR) + (-0.03589435 × BSPRY) + (0.19647862 × CCNC) + (0.05115348 × NSMCE1) + (0.08583657 × OTUB1) + (0.10935190 × PJA1) + (0.20917433 × RNF10) + (0.18285566 × RNF14) + (0.06215722 × RNF149) + (0.22512259 × RNF181) + (0.03997691 × UBE2D2) + (0.02151761 × USP10) + (-0.22326703 × USP9Y). The risk score distribution analyses and survival status illustrated a higher risk of death in the high-risk group (Fig. **5B**). Kaplan-Meier analysis showed that low-risk patients had a better prognosis (*p*=1.9e-09, Fig. **5C**), and the operating characteristic curve showed that the model was feasible (Fig. **5D**). Subsequent univariate and multivariate Cox regression analyses with 95% confidence intervals confirmed that age, tumor stage, and the calculated risk score emerged as significant independent prognostic indicators for NPC (*p*<0.05, Figs. **5E**, **F**).

In an effort to enhance the prognostic prediction for NPC, a predictive nomogram was constructed incorporating five independent prognostic factors identified through a Cox proportional hazards regression model analysis. The nomogram demonstrated that the risk score possessed the highest area under the curve, with age and disease stage ranking subsequent in predictive accuracy (Fig. **5G**). To assess the predictive accuracy of the nomogram, calibration curves were meticulously constructed. The calibration plots corresponding to the 1-, 3-, and 5-year OS probabilities of the model closely aligned with the reference line, thereby attesting to the outstanding predictive performance of the risk assessment model (Fig. **5H**). Finally, the decision curve analysis was used to determine the clinical utility of the nomogram, and the model had excellent performance in predicting the OS probability (Fig. **5I**). Upon examination of the risk scores within the two distinct cluster groups, it was determined that the URGcluster_B cohort exhibited a significantly elevated risk score compared to its counterpart (Fig. **5J**). The Sankey Figure shows that most of the samples in cluster B belong to high-risk, and most of the samples in URGcluster_A belong to low-risk (Fig. **5K**).

To assess the robustness of the URG-based risk model, we conducted an external validation using the GSE102349 NPC cohort. Patients were divided into high-risk and low-risk groups based on the median risk score. The distribution of patient risk scores was plotted alongside their survival status (Supplementary Fig. **3A**). The results demonstrated that patients with higher risk scores exhibited shorter progression-free survival. Kaplan-Meier survival analysis revealed a statistically significant difference in PFS between the two groups, with high-risk patients exhibiting poorer survival outcomes (*p*=0.027, Supplementary Fig. **3B**). The AUC of the ROC curve confirmed the prognostic value of the risk model in distinguishing high-risk from low-risk patients (Supplementary Fig. **3C**). These findings provide independent validation of the URG-based risk model's prognostic utility, reinforcing its potential clinical applicability in NPC.

Taken together, the risk model has excellent prognostic predictive value in NPC patients.

### 3.4 Immunotherapy Prediction for URG-based Risk Model

Subsequently, the Tumor Immune Dysfunction and Exclusion (TIDE) algorithm, an online analytical tool, was employed to prognosticate the efficacy of immune checkpoint therapy among cancer patients [24, 25]. Our analysis revealed that the TIDE score was markedly elevated in the high-risk group relative to the low-risk group, indicating a significant difference in the risk assessment metric between the two cohorts (Fig. **5L**). Moreover, TIDE analysis predicted the probability of response to immune checkpoint inhibitors in different groups. In contrast to the high-risk cohort, patients categorized within the low-risk group exhibited a significantly higher magnitude of response to immunotherapy, indicating a pronounced therapeutic benefit (Fig. **5M**). These results confirmed that the risk model has an excellent predictive value for the outcome of immunotherapy in NPC patients.

### 3.5 Validation of URG-based Risk Model Genes in the TCGA Dataset

**Fig. (6):**
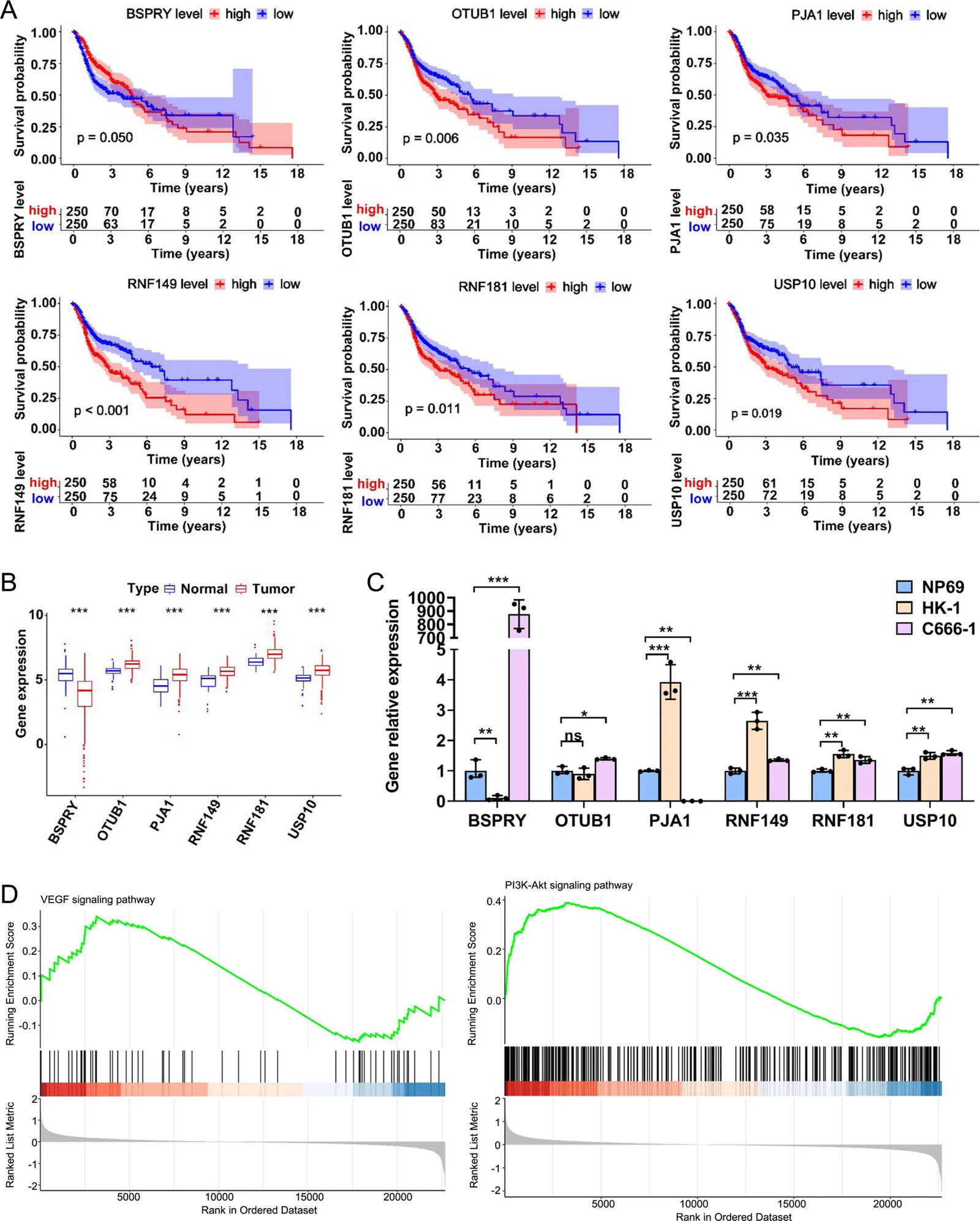
Validation of risk model URGs. (**A**) Survival analysis of 6 model URGs by Kaplan-Meier. (**B**) Expression levels of 6 model URGs in TCGA database. (**C**) The qPCR detection of 6 candidates URGs expressed in NPC cells. NP69 was served as control. (**D**) GSEA pathway in NPC tissues including VEGF signaling pathway and PI3K-AKT signaling pathway. *** *p* < 0.001, ** *p* < 0.01, * *p* < 0.05.

We validated the expression of 13 risk model URGs in TCGA-HNSC cancer and paracancerous samples, but only 6 URGs (BSPRY, OTUB1, PJA1, RNF149, RNF181, USP10) exhibited expression patterns consistent with their prognostic trends (*p* < 0.05, Figs. **6A**-**B**). Among them, except for BSPRY, the other five genes were significantly upregulated in tumor tissues and were associated with poor prognosis in patients. We further validated the expression of these 6 candidate URGs in NPC cells using qPCR. However, the results revealed that only RNF149, RNF181, and USP10 exhibited expression trends fully consistent with the TCGA findings. Compared to normal epithelial cells (NP69), the expression of RNF149, RNF181, and USP10 was significantly upregulated in NPC/HK-1 and NPC/C666-1 cells (Fig. **6C**). Given that RNF149 is an E3 ubiquitin ligase and exhibits the most significant intergroup differences, we selected RNF149 for further analysis. GSEA analysis showed that the high expression of RNF149 was mainly enriched in the VEGF signaling pathway and PI3K-Akt signaling pathway (Fig. **6D**). In short, we subsequently chose to focus on RNF149 for functional validation.

### 3.6 RNF149 Promotes NPC Progression *in Vivo* and *in Vitro*

**Fig. (7):**
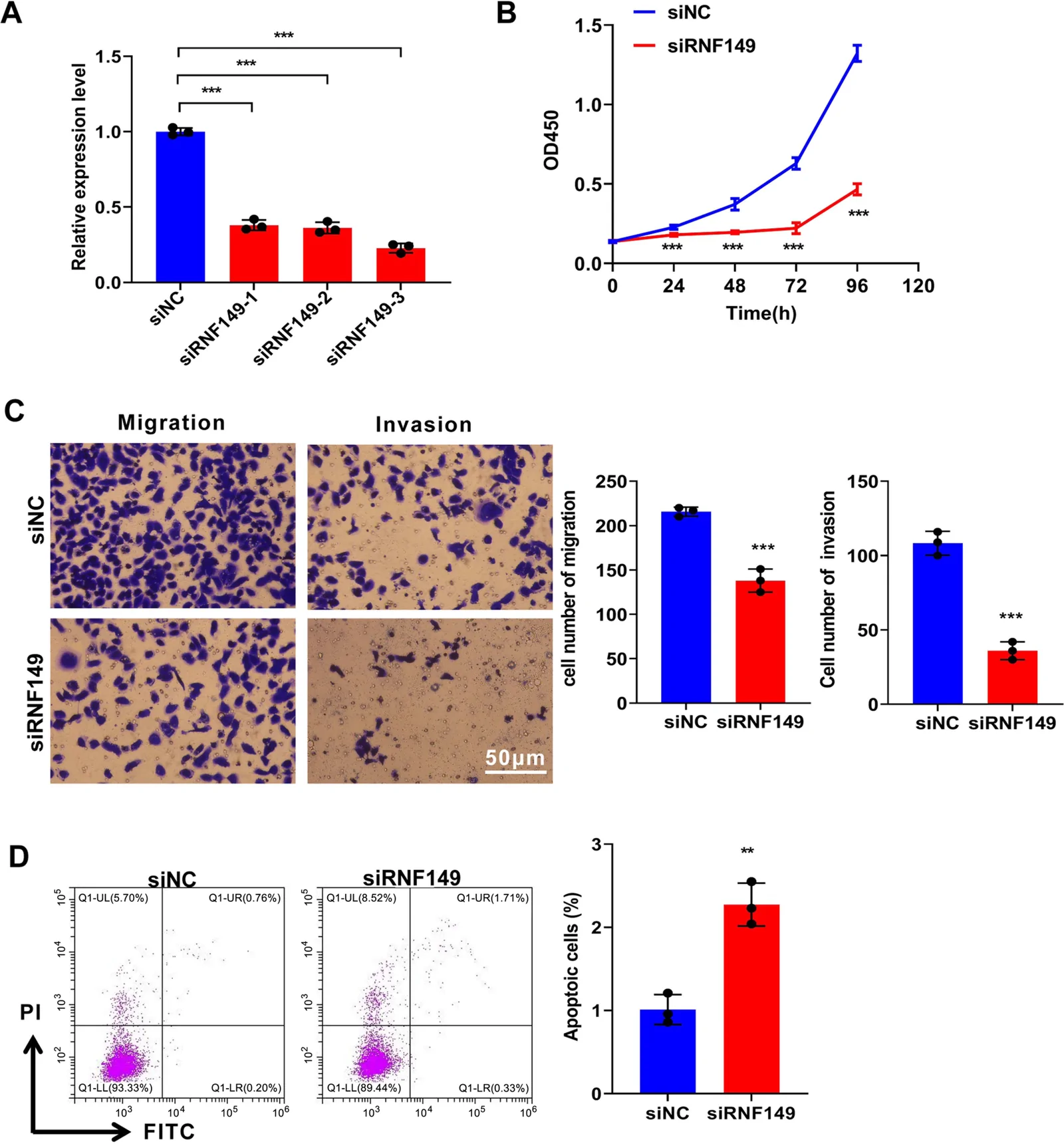
Functional phenotype of RNF149 in NPC cells. (**A**) Detection of the siRNA efficiency of knockdown RNF149 in HK-1 cells. (**B**) CCK-8 assay was used to detect cell proliferation of HK-1 cells after interference of RNF149. (**C**) Transwell assay was used to detect cell migration and invasion of HK-1 cells after interference of RNF149. Scale bar, 80 µm. (**D**) Flow cytometry was used to detect apoptotic cells of HK-1 cells after interference of RNF149. *** *p* < 0.001.

To investigate the effect of RNF149 on NPC, we silenced RNF149 expression in NPC/HK-1 cells and subsequently assessed cellular functions. The results demonstrated that siRNA effectively reduced RNF149 mRNA expression levels (Fig. **7A**). The results revealed that knockdown of RNF149 significantly impaired the proliferation, migration, and invasion capabilities of NPC/HK-1 cells (Figs. **7B**, **C**). Conversely, the apoptosis levels of NPC/HK-1 cells were markedly increased following RNF149 silencing (Fig. **7D**). Our findings demonstrate that RNF149 knockdown significantly impeded the proliferative, migratory, and invasive capabilities of NPC cells.

**Fig. (8):**
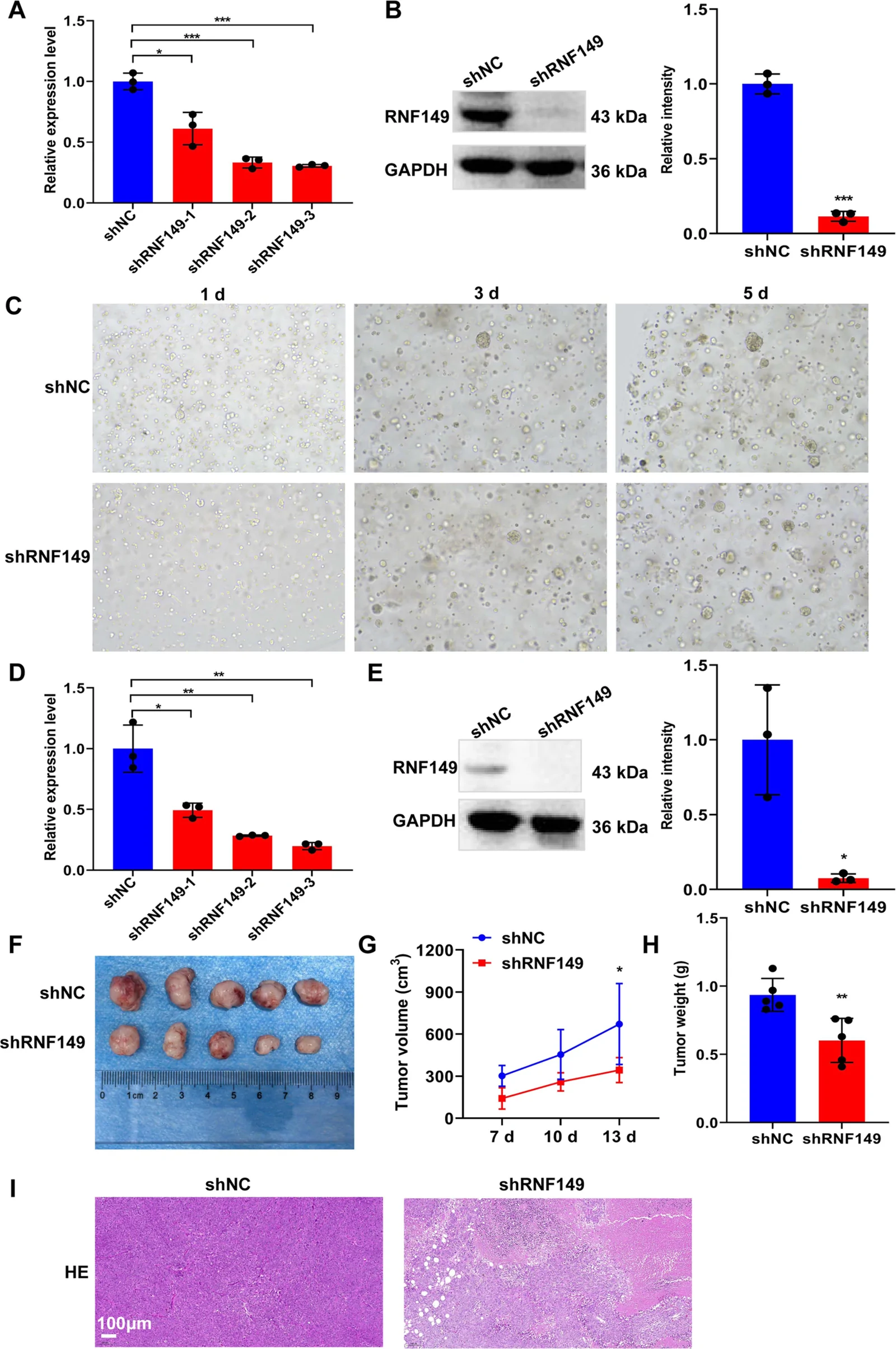
RNF149 promotes NPC progression *in vivo*. (**A**) The mRNA expression efficacy of RNF149 in NPC organoids was detected by qPCR. (**B**) The protein expression efficacy of RNF149 in NPC organoids was detected by Western blot. (**C**) Representative images of NPC organoids in shNC group and shRNF149 group at different time points. (**D**) The mRNA expression efficacy of RNF149 in HK-1 cells was detected by qPCR. (**E**) The protein expression efficacy of RNF149 in HK-1 cells was detected by Western blot. (**F**) Images of *ex vivo* tumor tissues along with statistical graphs of tumor volume (**G**) and weight (**H**), n=5. (**I**) HE staining of tumor tissues in two groups. Scale bar, 100 µm.*** *p* < 0.001.

Additionally, tumor organoid and tumor-bearing mice were established to verify the role of RNF149. After enzymatic digestion, NPC organoids were successfully induced and established from tumor tissues derived from five patients with NPC (Table **1**). Among the tested shRNAs, shRNA-3 exhibited the highest knockdown efficiency of RNF149 and was thus selected for the formal organoid construction experiments (Figs. **8A** and **8B**). As expected, the RNF149 knockdown group exhibited a reduced capacity to form NPC organoids in a 3D culture system (Fig. **8C**). The HK-1 cells stably transfected with shRNA-3 exhibited the significant knockdown efficiency of RNF149 at both the mRNA and protein levels (Figs. **8D** and **8E**). Consistent with the findings observed in organoids, we found that the subcutaneous tumorigenic capacity of HK-1 cells stably transfected with shRNA-RNF149 was diminished compared to the control group, as evidenced by significantly reduced tumor volume and weight (Figs. **8F** - **8H**). H&E staining further confirmed the pathological characteristics of tumor tissues in both groups (Fig. **8I**). Collectively, the knockdown of RNF149 expression inhibits NPC tumor growth.

## 4 DISCUSSION

NPC is a squamous cell cancer and the diagnosis of NPC has made significant progress, while many patients still have a poor prognosis. Ubiquitination modification is important in cancer development and progression [26, 27]. It’s generally believed that gene regulation plays a key role in tumors, and ubiquitination is an important factor [28, 29]. Here, we analyzed the gene expression profiles of URGs in NPC. Our study identified 33 differentially expressed URGs as signature genes. By identifying and analyzing URGs, we developed a risk model that can be used to predict the prognosis of individual NPC patients with high accuracy. The risk model can provide individualized evaluation for the treatment and prognosis of NPC, and its predictive ability for OS is better than other factors. Several studies have reported that PJA1 of the PRAJA family of E3 ubiquitin ligases plays a role in a variety of diseases. For example, OTUB2 promotes liver cancer cell proliferation and migration through PJA1 deubiquitination [30]. Luo *et al.* found that PJA1 regulated apoptosis and invasion of lung adenocarcinoma by promoting FOXR2 degradation [31]. Among E3 ubiquitin ligases, the RING-finger (RNF) family is the largest, which mediates ubiquitination through its RING-finger domain [32]. RNF149 is located on human chromosome 2 and mutated in several human tumors. Guo *et al.* found that RNF149 promotes the progression of hepatocellular carcinoma through E3 ubiquitin ligase activity [17]. In our study, PJA1 and RNF149 genes were significantly different among the risk model genes, and their expression was consistent with the prognostic trend. In addition, we found that PJA1 was associated with oncogenic function but negatively correlated with immune function, and knockdown RNF149 inhibited the proliferation of NPC cells. Similar studies have also been reported. In the glioblastoma model, PJA1 knockdown increased the stability of capicua and extended the survival period [33]. Another study found that MFSD4A could specifically bind and degrade EPHA2 by recruiting RNF149 and alter the epithelial-mesenchymal transition mediated by EPHA2, thus affecting the progression of NPC [34]. Bulk RNA-seq mainly focuses on the average expression level of all cells, while scRNA-seq can identify the heterogeneity within tumors at the cellular level, and many novel cancer biomarkers have been identified through combined analysis [35, 36]. Here, we identified URGs and identified URGs-related subtypes with completely different tumor immune microenvironment. We subsequently constructed a URG-based prognostic risk model for NPC that demonstrated superior predictive accuracy (AUC = 0.709) compared to the previously reported biomarkers such as tumor-educated platelet lncRNA ROR (AUC = 0.70) [37], 3-miRNA signature (AUC = 0.69) [38], and six-consensus-factor model (AUC = 0.674) [39]. In addition, we identified PJA1 and RNF149 as core genes associated with ubiquitination for NPC prognosis, which may be a potential marker for NPC, which is worth further research. Overall, the stratification of NPC patients according to the risk model, and then exploring the sensitive treatment of high-risk patients, will help physicians to different risk stratification of patients make reasonable clinical decisions, individualize treatment actively, to improve the prognosis.

At the same time, we also revealed immunotherapy responses between high-risk and low-risk groups. These confirmed the risk model had an excellent predictive effect on the prognosis of NPC immunotherapy. NPC is characterized by a highly immunosuppressive tumor microenvironment, which plays a critical role in immune evasion and resistance to immunotherapy. Several mechanisms contribute to immune evasion in NPC, including upregulated expression of immune checkpoint molecules (*e.g.*, PD-L1, CTLA-4) [40], expansion of immunosuppressive regulatory T cells [41], tumor-associated macrophages [42], and myeloid-derived suppressor cells [43], all of which contribute to T-cell exhaustion and reduced anti-tumor immunity. Additionally, Epstein-Barr virus (EBV)-associated NPC exhibits unique immunological features, including viral antigen-induced immune tolerance and chronic inflammation, further hindering effective immune responses [44]. In this study, our URG-based risk model effectively stratified NPC patients into high- and low-risk groups, with the low-risk group demonstrating a significantly improved response to immunotherapy. This suggests that the genes incorporated in our model may reflect underlying immune activity within the tumor microenvironment, potentially influencing the effectiveness of immune checkpoint blockade therapies. Patients in the high-risk group, who exhibit poorer immunotherapy responses, may have tumors with stronger immune evasion mechanisms, emphasizing the need for alternative therapeutic strategies such as combination therapies to enhance treatment efficacy. By identifying patients who are more likely to benefit from immunotherapy, our model provides a valuable tool for risk stratification and treatment decision-making. Future studies should further explore the molecular pathways linking ubiquitination-related genes to immune regulation in NPC to optimize immunotherapy strategies.

Despite the promising findings of this study, several limitations should be acknowledged. First, although the scRNA-seq dataset provided valuable insights into the differential expression of URGs in NPC, the dataset size was limited, and further validation using larger, independent cohorts is required to confirm the robustness of our risk model. Moreover, while scRNA-seq provides high-resolution insights into tumor heterogeneity, its widespread clinical adoption is limited by technical constraints, including high sequencing costs, the need for specialized bioinformatics expertise, and batch effects that may influence data reproducibility the transition from scRNA-seq-derived biomarkers to clinically feasible assays, such as qPCR or immunohistochemistry, requires validation to confirm their diagnostic and prognostic accuracy in clinical samples. Second, the functional characterization of RNF149 in NPC was primarily conducted *in vitro* and in subcutaneous tumor-bearing mouse models. Given that NPC is known for its unique anatomical location and propensity for lymphatic metastasis, future studies should employ orthotopic or metastatic models to better recapitulate the tumor microenvironment and disease progression. Moreover, while our results demonstrated that RNF149 knockdown attenuates tumor growth and impairs organoid formation, the underlying mechanisms driving these effects remain to be fully elucidated. In particular, the specific downstream targets and signaling pathways regulated by RNF149 in NPC warrant further investigation to better understand its oncogenic role. From a therapeutic perspective, targeting RNF149 holds potential as a novel strategy for NPC treatment. The significant reduction in tumorigenic capacity following RNF149 knockdown suggests that it may serve as a viable target for molecular therapies. However, the development of RNF149 inhibitors or targeted degradation strategies remains in its infancy, and further studies are needed to evaluate their efficacy and safety in preclinical and clinical settings. Additionally, given the role of ubiquitination in immune regulation, and RNF149 is a URG, it is worth exploring whether RNF149 inhibition could enhance the efficacy of immunotherapy by modulating the tumor immune microenvironment. Overall, while our findings provide compelling evidence for RNF149 as a potential therapeutic target in NPC, future research should focus on addressing these limitations and advancing translational applications.

## CONCLUSION

In conclusion, our research has delineated the presence of unique URGs in NPC patients. We have successfully developed and validated a URGs-related prognostic risk model capable of predicting clinical outcomes in NPC patients. Notably, patients classified into the low-risk group exhibited a significantly enhanced response to immunotherapy, highlighting the potential clinical utility of our model in guiding personalized treatment strategies. Furthermore, we experimentally confirmed that RNF149 promotes NPC progression *in vitro*, organoid, and *in vivo*, supporting its role as a potential therapeutic target. These findings provide novel insights into the role of URGs in NPC pathogenesis and offer a foundation for risk stratification and targeted therapeutic interventions, ultimately contributing to improved patient prognosis.

## Figures and Tables

**Fig. (1):**
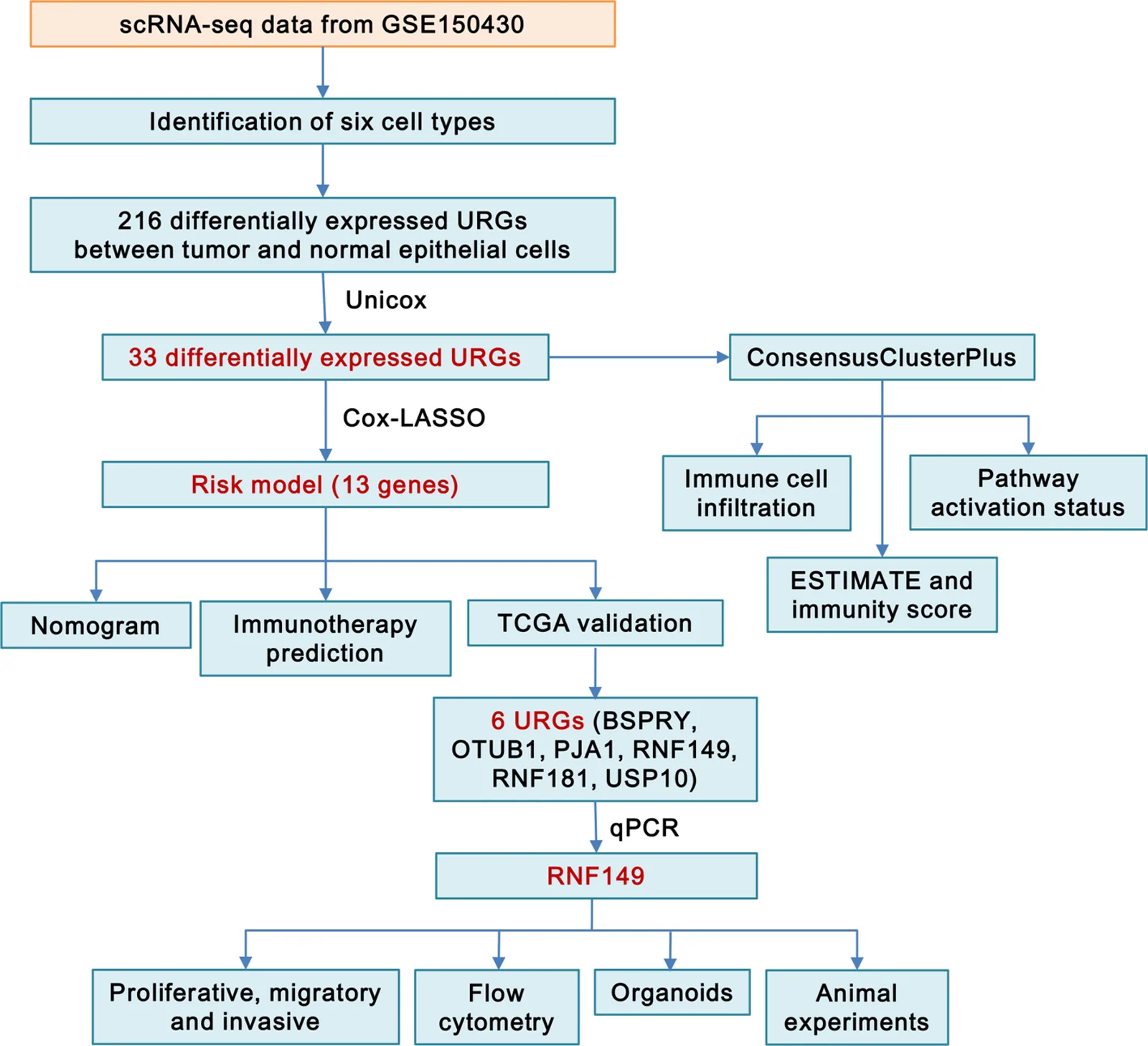
Flowchart of the research. URGs: ubiquitination-related genes.

**Table 1:** Clinical information for the study participants.

**Characteristics**	**Patient 1**	**Patient 2**	**Patient 3**	**Patient 4**	**Patient 5**
Age (year)	64	27	52	44	54
Gender	Female	Female	Female	Male	Female
Clinical staging	II	II	III	II	II
T stage	3	1	4	3	2
N stage	2	2	2	2	2
Distant metastasis (at diagnosis)	NO	NO	NO	NO	NO
Bone metastasis	NO	NO	NO	NO	NO

## Data Availability

All data generated or analyzed during this study are included in this published article.

## LIST OF ABBREVIATIONS

NPC: Nasopharyngeal Carcinoma
URGs: Ubiquitination Related Genes
NPC: Nasopharyngeal Carcinoma
OS: Overall Survival
GEO: Gene Expression Omnibus
NPC: Nasopharyngeal Carcinoma
EBV: Epstein-Barr Virus
PFS: Progression-free Survival
