# Supplementary material for: A Ubiquitination-Related Gene Prognostic Signature and the Oncogenic Role of RNF149 in Nasopharyngeal Carcinoma: scRNA-seq-Based Bioinformatics and Experimental Validation
Source: Curr Med Chem. 2025 Jun 25;33(21):4259–81. doi: 10.2174/0109298673378818250610053439 (PMC13555840; doi:10.2174/0109298673378818250610053439)
Supplement: Supplementary file 1 [file CMC-33-21-4259_SD1.pdf]

Supplementary Material

A Ubiquitination-Related Gene Prognostic Signature and the Oncogenic Role of RNF149 in Nasopharyngeal Carcinoma: scRNA-seq-Based Bioinformatics and Experimental Validation

Haiyan Deng[1,#,] Juan Zhang[2,#], Shuaijun Chen[2,#], Tingfeng Liang[2], Xueyong Hu[2], Jing Li[2], Yong He[2], Feng Yu[1,*] and Chaosheng Yu[1,2,*]

[1]Department of Otorhinolaryngology-Head and Neck Surgery, Guangzhou Red Cross Hospital of Jinan University, Guangzhou, Guangdong, China; [2]Department of Otorhinolaryngology-Head and Neck Surgery, Zhujiang Hospital, Southern Medical University, Guangzhou, China

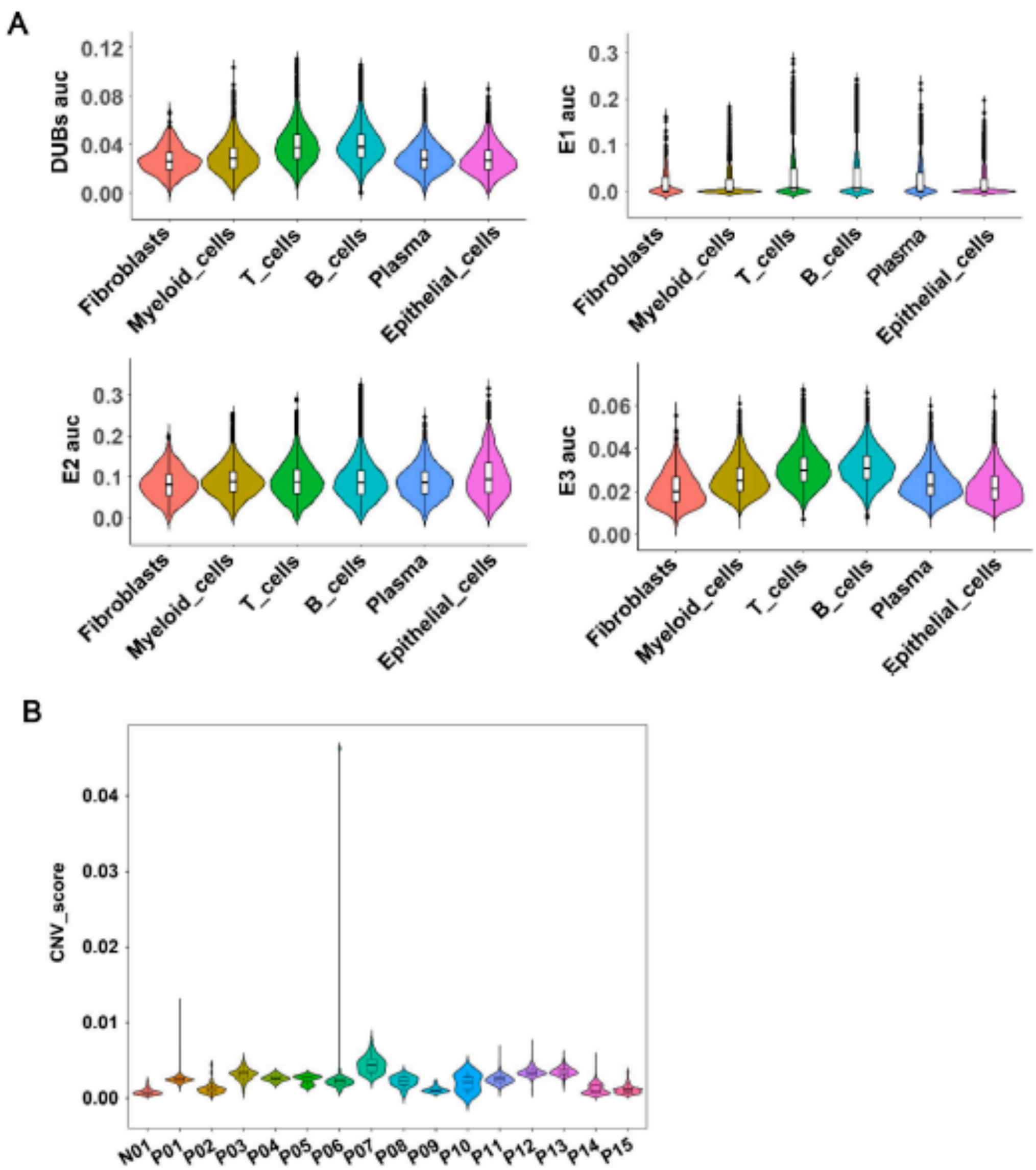

Fig. (S1). Comparison of scores in cell subtypes. (**A**) Scores of E1, E2, E3 and deubiquitinating enzymes (DUBs) in Fibroblasts, Myeloid_cells, T_cells, B_cells, Plasma, Epithelial_cells. (**B**) CNV score analysis of epithelial cells from all samples.

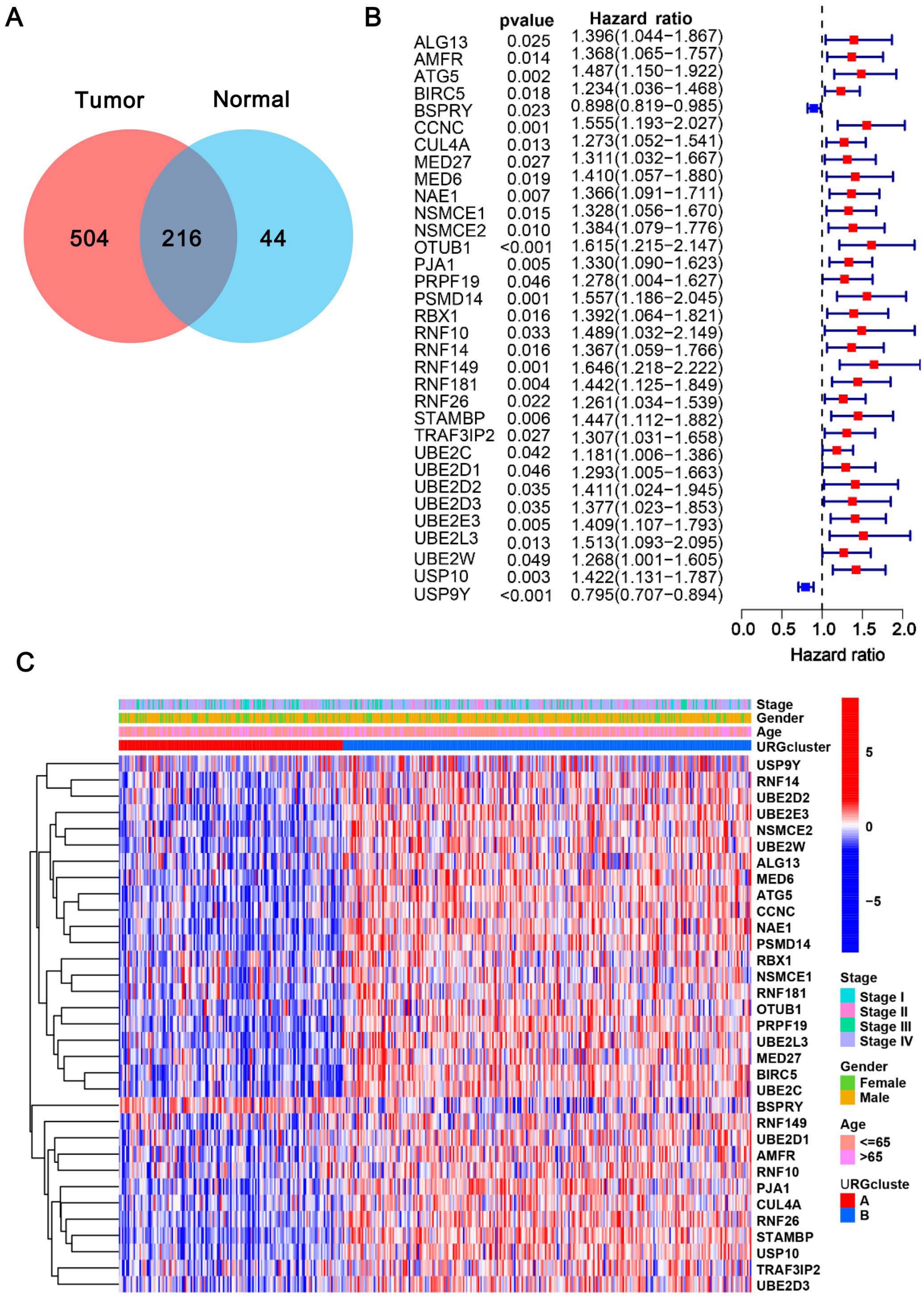

Fig. (S2). Screening of differentially expressed URGs. (**A**) DEGs between tumor and normal epithelial cells. (**B**) By univariate Cox regression analysis, 33 differentially expressed and highly mutated URGs associated with NPC were screened out. Parentheses indicate the 95% confidence intervals for the hazard ratios. (**C**) Heatmap of the expression of 33 URGs.

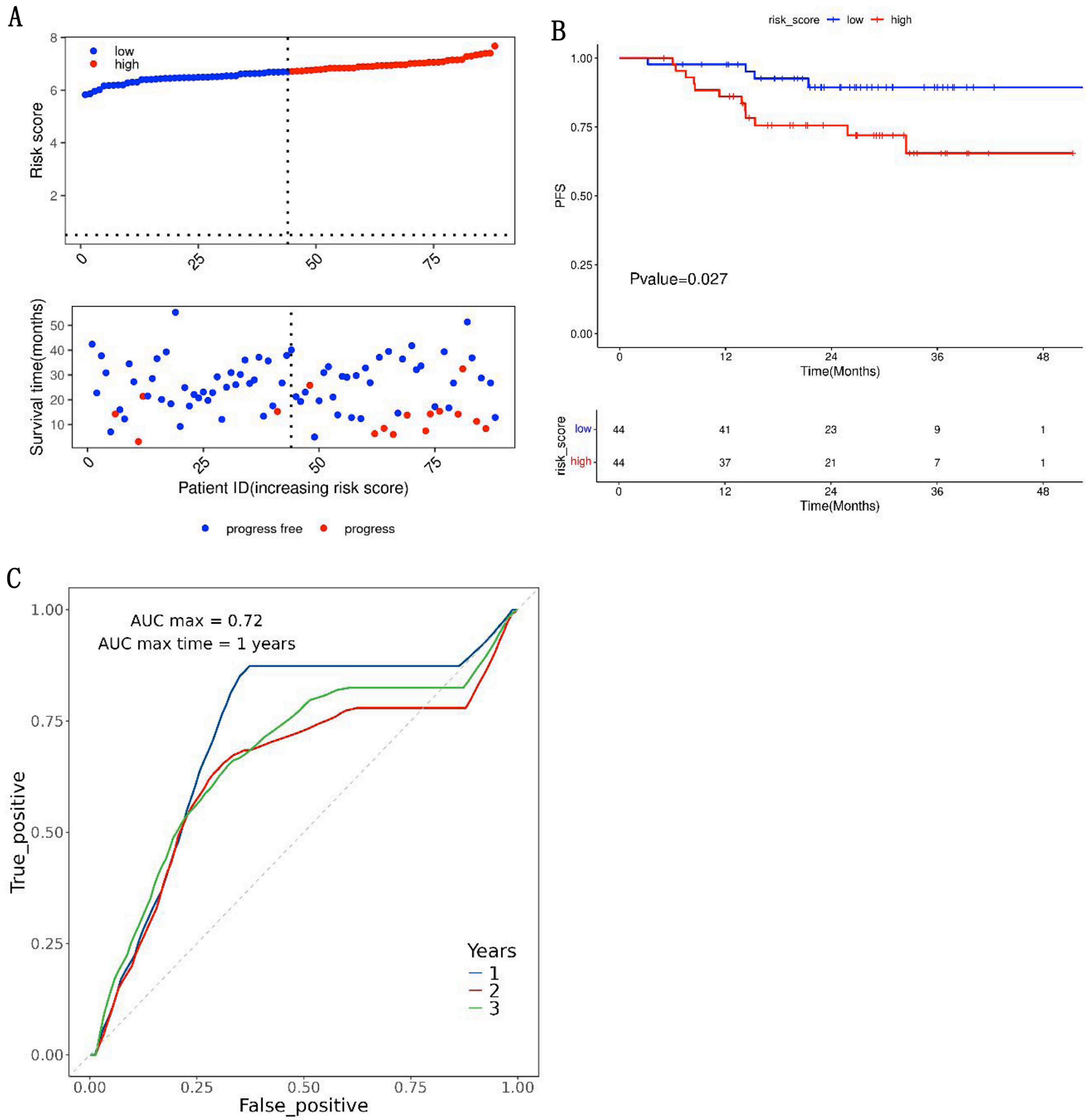

Fig. (S3). External validation of the prognostic risk model using GSE102349 NPC cohort. (**A**) Scatter plot illustrating the distribution of risk scores and survival status in the GSE102349 NPC cohort. Each dot represents a patient (n=88), with risk scores sorted in ascending order. Red dots indicate patients with disease progression, while blue dots represent patients without progression. (**B**) Kaplan-Meier survival curves for progression-free survival (PFS) in the GSE102349 cohort. (**C**) Receiver operating characteristic (ROC) curve assessing the predictive accuracy of the risk model in the GSE102349 cohort. The area under the curve (AUC) value indicates the model's ability to distinguish between high-risk and low-risk patients, demonstrating its prognostic utility in an independent dataset.

Table S1. The sequences of qPCR primers.

Gene	Primers (5' - 3')
GAPDH-F	ACAACTTTGGTATCGTGGAAGG
GAPDH-R	GCCATCACGCCACAGTTTC
BSPRY-F	CCTGCCGGGGAAGAATCAAAG
BSPRY-R	AGACTCAACCGCTGGATAACC
OTUB1-F	TCGGTCCTATACAAGGAGTATG
OTUB1-R	GGTCTTGCGGATGTACGAGT
PJA1-F	CTATCCCGAGCCAAAGTACCC
PJA1-R	TCAGAGTCTTCGTCGCAGTATT
RNF149-F	AACATCACCTTGCCCATGTCT
RNF149-R	CCCCTATGGTCATCGTTACTGG
RNF181-F	CCCTGGCTAAGCAAGACAAAT
RNF181-R	TGCTCCTCATAAGTGTCGTCAT
USP10-F	AGGCTGTGGATAAACTACCTGAT
USP10-R	GCTTGAAATACTGTAGCTGGGG
si-NC	UUCUCCGAACGUGUCACGUTT<br>ACGUGACACGUUCGGAGAATT
siRNF149-3	CAGGAAAUAUAGUGGUCAUUA<br>AUGACCACUAUAUUUCCUGUU
siRNF149-2	GGAAUUCCAGUAACGAUGACC<br>UCAUCGUUACUGGAAUUCCUU
siRNF149-1	GAAUUGAUGUUGAUGCUGAAATT<br>UUUCAGCAUCAACAUCAAUUCTT
